# Immunogenicity analysis of genetically conserved segments in *Plasmodium ovale* merozoite surface protein-8

**DOI:** 10.1186/s13071-019-3412-0

**Published:** 2019-04-11

**Authors:** Xinxin Zhang, Ruilin Chu, Sui Xu, Haitian Fu, Jianxia Tang, Limei Chen, Xiaodan Shi, Jing Chen, Yuhong Li, Guoding Zhu, Eun-Taek Han, Yinghua Xuan, Jun Cao, Yang Cheng

**Affiliations:** 1 Laboratory of Pathogen Infection and Immunity, Department of Public Health and Preventive Medicine, Wuxi School of Medicine, Jiangnan University, Wuxi, Jiangsu People’s Republic of China; 2 Key Laboratory of National Health and Family Planning Commission on Parasitic Disease Control and Prevention, Jiangsu Provincial Key Laboratory on Parasite and Vector Control Technology, Jiangsu Institute of Parasite Diseases, Wuxi, Jiangsu People’s Republic of China; 3 Department of Medical Environmental Biology and Tropical Medicine, School of Medicine, Kangwon National University, Chuncheon, Gangwon-do Republic of Korea

**Keywords:** *Plasmodium ovale*, MSP-8, Immunogenicity, Cross-reactivity, Conservation

## Abstract

**Background:**

*Plasmodium ovale* is widely distributed across tropical regions and has two closely related but distinct species, namely *P. ovale curtisi* and *P. ovale wallikeri*. Combining genetic characterization with the immunogenicity of merozoite surface protein-8 (MSP-8) supports considering MSP-8 as a candidate target for blood-stage vaccines against malaria. However, no previous studies have focused on characterizing the genetic diversity and immunogenicity of PoMSP-8.

**Methods:**

Blood samples were collected from 42 patients infected with *P. ovale*. The patients were migrant workers returning to the Jiangsu Province from Africa; genomic DNA was extracted from their blood samples for sequencing and protein expression. The recombinant PoMSP-8 (rPoMSP-8) proteins were expressed and purified to assess their immune responses in BALB/c mice.

**Results:**

The sequences of the *P. ovale curtisi* and *P. ovale wallikeri msp8* genes were completely conserved in each isolate. The rPoMSP-8 proteins were successfully expressed and purified as ~70 kDa proteins. Antibodies raised against rPoMSP-8 in mice showed appropriate immunoreactivity, as evidenced by immunoblotting. These specific antibodies were detected at day 7 post-immunization, and their levels increased throughout the whole immunization period. rPoMSP-8-raised antibodies had high endpoint titers (1:5,120,000) and high avidity (PocMSP-8: 94.84%, PowMSP-8: 92.69%). Cross-reactivity between rPocMSP-8 and rPowMSP-8 was observed with each anti-PoMSP8-specific antibody.

**Conclusions:**

Remarkable conservation and high immunogenicity was observed in both rPoMSP-8s. Intriguingly, cross-reaction between rPocMSP-8 and rPowMSP-8 was detected, suggesting that a single PoMSP8-based construction might be applicable for both species.

**Electronic supplementary material:**

The online version of this article (10.1186/s13071-019-3412-0) contains supplementary material, which is available to authorized users.

## Background

Malaria remains a disease of global health importance [1]. In 2017, an estimated 219 million cases of malaria and 435,000 malaria-caused deaths were reported [2]. One causative agent of human malaria, *Plasmodium ovale*, was first described by Stevens [3]. Ovale malaria in humans is caused by two closely related but distinct subspecies of the malarial parasite, *P. ovale curtisi* (the classic type) and *P. ovale wallikeri* (the variant type) [4]. Little attention has been paid to ovale malaria because it often presents with low parasitemia and mixed infection with other *Plasmodium* species, with mild clinical symptoms that are easily treated with the conventional antimalarial drug chloroquine [5–9].

Jiangsu Province, located in eastern China, was previously an unstable malaria-transmission area. After large-scale implementation of interventions such as mass drug administration, indoor residual spraying, and long-lasting insecticide nets, malaria has been effectively controlled in the Jiangsu Province [10], and no report of local malaria infection has been issued since 2012 [11]. With the continuous expansion and deepening of the China-Africa cooperation, large numbers of Chinese people travel to malaria-endemic countries for trade, tourism, labor and other purposes [12]. Between 2014 and 2016, 1068 imported cases of malaria were reported in the Jiangsu Province, and approximately 170 cases of malaria in China imported from Africa were caused by *P. ovale* [13].

Parasite antigenic diversity has become a major obstacle hindering the development of effective malaria vaccines [14]. Erythrocyte invasion is a critical point in the life-cycle of malaria parasites and it is at this point that merozoites can be targeted by antibodies and eliminated by immune cells [15]. This type of vaccine generally incorporates antigenic proteins that are highly expressed on, or associated with, the surface of merozoites, such as the merozoite surface proteins (MSPs) [14]. MSPs comprise some of the best characterized proteins in *P. falciparum* and some of them are considered promising vaccine candidates due to their accessibility to interaction with host immune system molecules [15]. MSP-8 is a glycosylphosphatidylinositol (GPI)-anchored protein produced by blood-stage malaria parasites. MSP-8 contains a signal sequence at the N-terminus and two epidermal growth factor (EGF)-like domains at the C-terminus with significant homology to those of other *Plasmodium* species. Serum from patients infected with *P. falciparum* could react against several PfMSP-8 fragments [16]. Likewise, *P. vivax* MSP-8 could induce humoral and cellular immune responses during *P. vivax* infection [17]. These data demonstrated that both PfMSP-8 and PvMSP-8 were immunogenic during natural infection. Immunization with *P. yoelii* MSP-8 (PyMSP-8) induced a level of protection in mice to lethal *P. yoelii* challenge [18]. Thus, PyMSP-8 was a target of protective immune responses. MSP-8 proteins are highly conserved across all *Plasmodium* sp. [19]. The conservation of MSP-8 proteins support their consideration as part of malaria vaccine formulations [20].

Therefore, MSP-8 from various malarial parasite species have been considered good vaccine candidates. However, insufficient evidence exists regarding the diversity and immunogenicity of *P. ovale* MSP-8 (PoMSP-8). In this study, *msp8* sequences were analyzed from both *P. ovale* sp. obtained from infected migrant workers returning to China from Africa. Sequence analysis of the *pomsp8* genes showed complete conservation. The recombinant PoMSP-8 (rPoMSP-8) proteins were produced, and high immunogenicity was detected in immunized mice.

## Methods

### Study areas and sample collection

*P. ovale curtisi* and *P. ovale wallikeri* samples were obtained at local hospitals in the Jiangsu Province in China between 2012 and 2016 from febrile patients who had recently returned from working in tropical regions of sub-Saharan Africa, endemic for malaria [21]. Identification of the isolates was confirmed by polymerase chain reaction (PCR) analysis and *Plasmodium* species were distinguished by real-time TaqMan PCR [11]. A total of 42 *P. ovale-*infected blood samples were randomly selected and analyzed. As a result, we identified 24 *P. ovale curtisi* samples and 18 *P. ovale wallikeri* samples. Full details of the isolates were provided (Additional file 1: Table S1).

### Cloning and sequencing

The full-length sequences of *pocmsp8* and *powmsp8* were amplified by PCR using the primers *pocmsp8*-Forward (5′-ATG GTT ATG ATT ATG AAA AAG AA-3′) and *pocmsp8*-Reverse (5′-CTA TAA TAA ATA TAC ACA TAA CAT C-3′), and *powmsp8*-Forward (5′-ATG GTT ATG ATT ATG AAA AAG AA-3′) and *powmsp8*-Reverse (5′-CTA TAA TAA ATA TAC ACA TAA CAT C-3′), respectively. The *pocmsp8* (PlasmoDB, PocGH01_10040100) and *powmsp8* (GenBank accession number, SBT74417.1) sequences from the *Plasmodium* Genomics Resource database and the National Center for Biotechnology Information GenBank database were used as reference gene sequences. The reactions were performed in a 20 µl reaction volume, including 1 μl of genomic DNA, 7.4 μl of double-distilled water, 0.8 μl of each primer, 0.5 units of DNA polymerase, 2 mM deoxynucleoside triphosphate and 10 μl of premix (2× Phanta^®^ Max Master Mix, Vazyme, Nanjing, China). PCR amplification was performed in a Mastercycler (Eppendorf, Hamburg, Germany) with the following program: denaturation at 95 °C for 3 min; 35 cycles of 95 °C for 15 s, 45 °C for 15 s and 72 °C for 30 s; and a final extension at 72 °C for 5 min. The amplified products were analyzed by 1% agarose gel electrophoresis and visualized under an ultraviolet transilluminator (ChemiDoc MP, Bio-Rad, Hercules, CA, USA). The PCR product sizes were estimated based on the mobilities relative to a standard DNA marker (Transgen Biotech, Beijing, China). PCR products were cloned into the pUC57 vector and sequenced using universal primers (M13F: 5′-TGT AAA ACG ACG GCC AGT-3′; M13R: 5′-CAG GAA ACA GCT ATG AC-3′), which was performed by GENEWIZ (Suzhou, China) on an ABI 3730xl DNA Analyzer (Thermo Fisher Scientific, Waltham, MA, USA).

### Sequence alignment and data analysis

The primary structure of the PoMSP-8 protein was predicted with a bioinformatics tool (http://smart.embl-heidelberg.de/). To evaluate the diversity of both *P. ovale* sp., the *pocmsp8* and *powmsp8* sequences were used as templates and aligned using GeneDoc v.2.7.0.

### Recombinant protein expression and purification

The *pomsp8* gene were subcloned into the pET32a expression vector (YouLong Biotech, Shanghai, China). This vector adds thioredoxin and six-histidine tags at both the N- and C-terminal ends, enabling easier purification and immunodetection using monoclonal antibodies against the six-histidine tag. Next, *rosetta-gami* bacteria (Transgen Biotech) were transformed with the recombinant plasmids. This strain contains a plasmid that encodes additional copies of rare *Escherichia coli* (*E. coli*) tRNAs for enhanced expression of foreign proteins. Proteins were purified by YouLong Biotech. Protein expression was verified by 8% sodium dodecyl sulfate-polyacrylamide gel electrophoresis (SDS-PAGE) with Coomassie blue staining or western blot analysis.

### Mice immunizations

Female BALB/c mice, 6–8 weeks of age, were used for immunizations (rPoMSP-8-immunized group and the negative-control group, *n* = 4 per group). Purified rPoMSP-8, 30 μg, was injected into each mouse. Complete Freund’s adjuvant (CFA; Sigma, San Francisco, CA, USA) was used for the primary booster, and incomplete Freund’s adjuvant (IFA; Sigma, San Francisco, CA, USA) was used for the subsequent boosters. Boosters were administered on days 21 and 42 post-immunization. All injections were given intraperitoneally. The serum of each mouse was collected at days 0, 7, 14, 28, 35 and 49 post-immunization. Mice in the negative-control group were injected with a mixture of PBS and adjuvant.

For western immunoblot analysis, rPoMSP-8 antigens were electrophoretically transferred from SDS-PAGE gels to 0.2 μm polyvinylidene fluoride membranes (Millipore, Darmstadt, Germany) and probed with serum pooled from mice immunized with recombinant proteins acquired at day 49 and diluted 1:1000. Immunoreactivity was detected using a polyclonal goat anti-mouse antibody as a secondary antibody (Southern Biotech, Tuscaloosa, AL, USA).

### Antibody responses

Antibody responses to PoMSP-8 at 0, 7, 14, 28, 35 and 49 days after primary immunization were measured by performing enzyme-linked immunosorbent assays (ELISAs). Briefly, 50 ng of rPoMSP-8 antigens were coated overnight at 4 °C in 96-well ELISA plates (Corning, Corning, NY, USA). After blocking with 1× PBS containing 1% bovine serum albumin (BSA; Beyotime Biotechnology, Beijing, China), the serum samples were diluted 1:20,000 in 1× PBS containing Tween 20 (PBS-T) and 0.5% BSA, and incubated in duplicate wells for 90 min. Horseradish peroxidase-conjugated goat anti-mouse IgG antibodies (Southern Biotech) were diluted 1:5000 and used to detect the IgG antibodies bound to PoMSP-8. The reaction was developed by adding the 3, 3′, 5, 5′-tetramethylbenzidine (Invitrogen, Waltham, MA, USA) as a substrate and then stopped by adding 2 M H_2_SO_4_; the absorbance was then measured at 450 nm. To evaluate the titration of specific anti-PoMSP-8 antibodies, sera from mice in each group were pooled and used in serial dilutions (1:10,000 to 1:5,120,000) in ELISA.

The avidity of anti-rPoMSP-8 IgG antibodies was estimated as described previously [22]. Briefly, ELISA testing was performed as described above, except that the test was performed in duplicate plates. After diluting the sera (1:20,000) and incubating for 90 min, one of the plates was washed three times with PBS-T, and the other plate was incubated with the same sera and washed three times with dissociation buffer, comprised of PBS-T with 6 M urea. Then, the plates were washed once with PBS-T buffer. Incubation with a secondary antibody, the wash steps, and developing the enzyme reaction were performed as described above for the ELISAs. The avidity index (AI) for each sample was calculated as follows:

$$ {\text{AI}} = ({\text{OD}}_{ 4 50} \;{\text{of a sample treated with 6 M urea}}/{\text{OD}}_{ 4 50} \;{\text{of a sample not treated with 6 M urea}}) \times 100. $$

### Statistical analysis

Statistical analysis and graphing were done using GraphPad Prism software, v.5.0 (GraphPad Software Inc., San Diego, CA, USA). To analyze cross-reaction and antibody responses, the data were analyzed by SPSS v.16.0. Student’s t-test was used to analyzed data emerging on normal distribution, with probability (*P*) values > 0.05 considered to reflect not statistically significant differences. Phylogenetic trees for MSP-8 were constructed using the neighbor-joining method based on the nucleotide sequences. Phylogenetic relationships based on the *msp8* gene were estimated using the neighbor-joining (NJ) method in MEGA v.7.0, the general time reversible (GTR) nucleotide model with a gamma (γ)-distribution model of among-site rate variation and a proportion of invariable sites (i.e. the GTR + γ + I substitution model) determined. The evolutionary relationships of the aligned sequences were determined using MEGA v.7.0.

## Results

### Description and gene analysis of PoMSP-8

**Fig. 1:**
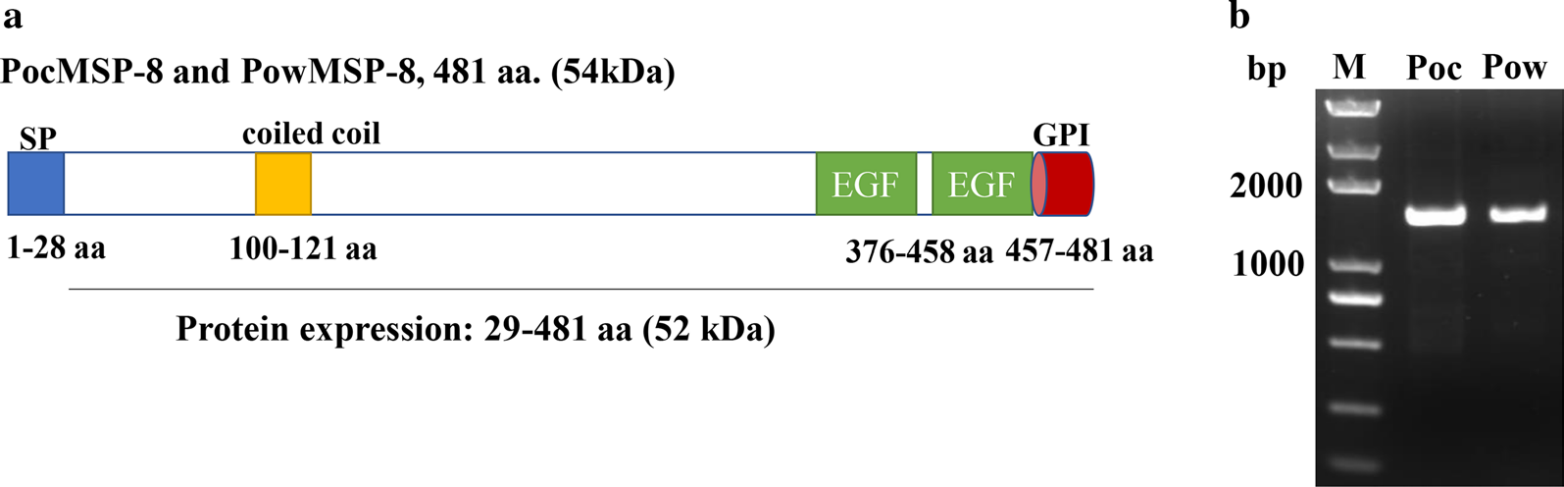
Schematic representation of the gene structure and amino acid sequence alignment of *P. ovale* MSP-8 C-terminal fragment. **a** Diagram of the gene structure of *pomsp*-8. Both *pomsp*-8 genes encode a signal peptide (blue), a coiled-coil domain (yellow), two EGF-like domains (green) and a GPI-anchor (red). **b** The full-length *msp*-8 genes of 42 *P. ovale* isolates were amplified by PCR

The single-exon *P. ovale curtisi* and *P. ovale wallikeri msp8* genes were both found to be 1446 base pairs long and to begin with a predicted 28-amino acid (aa) signal peptide sequence (aa 1–28). Some other specific regions were identified in the predicted primary structure of the *pomsp8*, such as a coiled-coil domain (aa 100–121), two epidermal growth factor (EGF)-like domains (aa 376–415 and 421–458), a transmembrane region (aa 461–480) and a GPI-anchor (aa 457–481) (Fig. 1a). To verify this putative gene structure for *pomsp*8, we prepared genomic DNA from patients infected with *P. ovale* and used it as the PCR template. *Pomsp8* genes were successfully amplified by PCR in all isolates, generating single PCR products of the expected size (~ 1.5 kb). Direct sequencing of these purified PCR fragments showed no superimposed signal on the electropherograms for *pomsp8* (Fig. 1b). Overall, the full length alignments for *pomsp8* showed that no amino acid mutations occurred among the 24 *pocmsp8* samples and 18 *powmsp8* samples originating from 15 countries in sub-Saharan Africa (Additional file 2: Figure S1). These findings suggested that the *pomsp8* genes were completely conserved in each isolate.

### Phylogenetic analysis

**Fig. 2:**
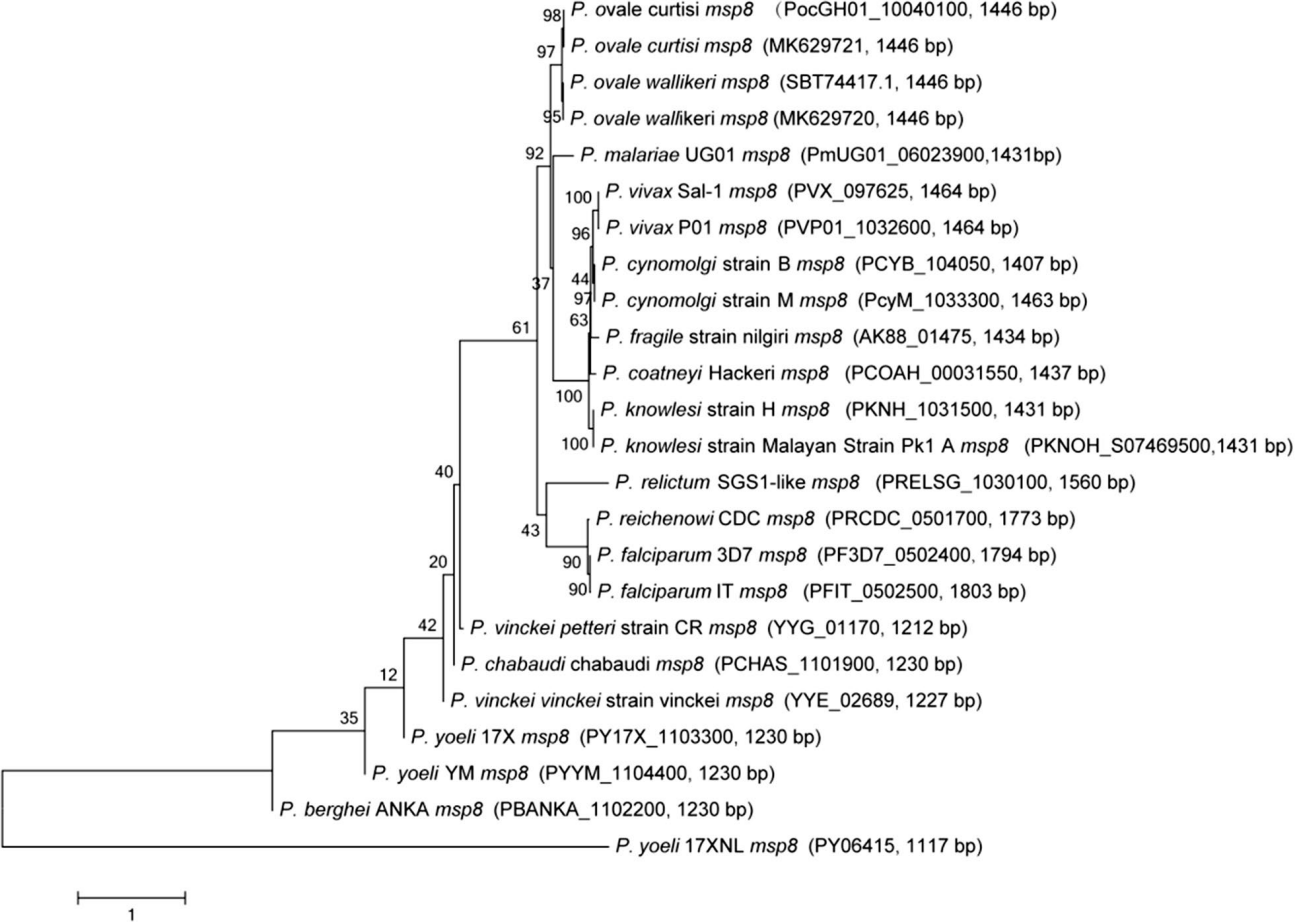
Phylogenetic trees using nucleotide sequences of the gene encoding MSP-8, based on the neighbor-joining method. The length of gene sequences was shown in the figure. The scale-bar indicates the number of nucleotide substitutions per site. The accession numbers of newly generated *pocmsp8* and *powmsp8* sequences were MK629721 and MK629720

Based on the high level of genetic conservation presented above, phylogenetic analysis was performed using *msp8* gene sequences from human, non-human primate, murine and avian malarial species, using the neighbor-joining method. This analysis showed that *pocmsp8* and *powmsp8* occupied a distinct bifurcating branch with 98 and 95% bootstrap support, respectively (Fig. 2). The *msp8* gene ID number of other *Plasmodium* species included in the analyses are provided in Additional file 3: Table S2.

### Expression and purification of recombinant PoMSP-8

**Fig. 3:**
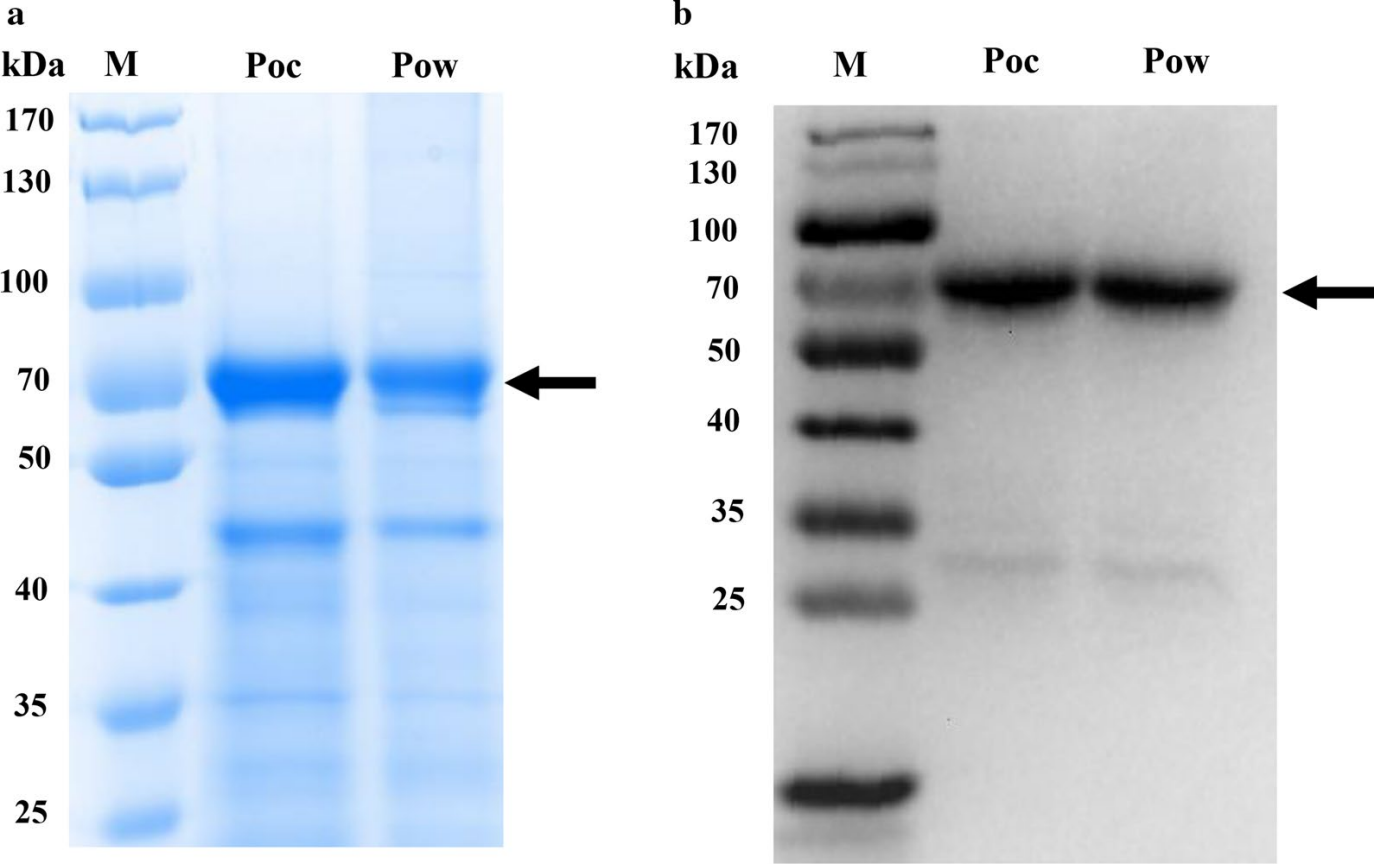
Expression and western blot analysis of PoMSP-8. **a** The purified PoMSP-8 proteins were analyzed by SDS-PAGE. Analysis of rPoMSP-8 showed that they had a molecular weight of approximately 70 kDa. **b** Western blot analysis of rPoMSP-8 using an anti-His antibody. Arrowheads indicate specific bands for each recombinant protein. A molecular mass marker is shown on the left-hand side in kDa

Truncated PoMSP-8 (ΔSP) proteins are predicted to consist of 430 aa residues with a molecular weight of 52 kDa. Both recombinant soluble forms of PoMSP-8 were successfully expressed using *E. coli* expression system and purified using an Ni-Sepharose column. The purity of the recombinant PoMSP-8 proteins were assessed. The pET32a adds thioredoxin and six-histidine tags at both the N- and C-terminal ends, so the proteins migrated with a molecular weight of ~70 kDa, based on SDS-PAGE analysis (Fig. 3a). Moreover, the corresponding immunoblots probed with an anti-His tag antibody confirmed that rPoMSP-8 was expressed (Fig. 3b). The confirmed proteins were used to immunize mice.

### Mice antibodies against PoMSP-8 recognized the recombinant proteins

**Fig. 4:**
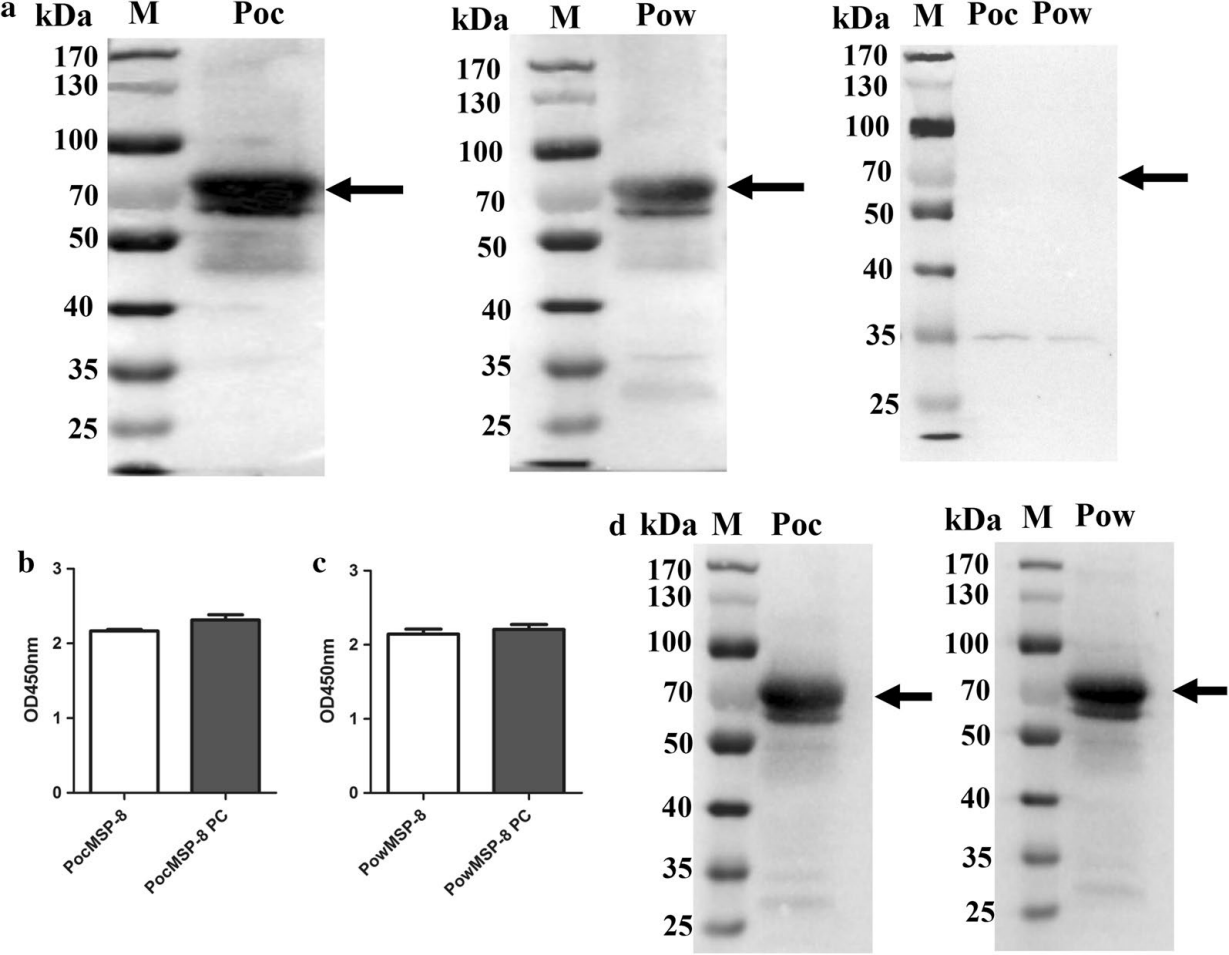
Reactivity of serum from mice immunized with rPoMSP-8. **a** Western blot analysis to determine whether the rPoMSP-8 proteins were reactive with sera from mice immunized with recombinant PoMSP-8 proteins, the sera from mice immunized with PBS and adjuvant as a negative control. **b** Cross-reaction of PocMSP-8 with sera from mice immunized with recombinant PowMSP-8 proteins. **c** Cross-reaction of PowMSP-8 with sera from mice immunized with recombinant PocMSP-8 proteins (PC, positive control). A statistically significant difference was not observed. **d** Cross-reactivity was verified by western blotting

To determine whether mice anti-rPoMSP8 antibodies could recognize the rPoMSP-8s, we developed an immunoblot for a specific ~70 kDa band that should be detected for both PoMSP-8s. The antibody responses of immunized mouse sera against PoMSP-8 were potent, suggesting that PoMSP-8 is highly immunogenic in mice. As a negative control, no reactivity was observed in sera from mice before immunization (Fig. 4a). To identify cross-reaction between PocMSP-8 and PowMSP-8, the antibodies raised against PowMSP-8 were reacted with rPocMSP-8, and mouse antiserum raised against PocMSP-8 were cross-reacted with rPowMSP-8 (Fig. 4b, c, *P* > 0.05). Then, cross-reactivity was verified using anti-rPoMSP-8 immune mouse serum with rPoMSP-8s (Fig. 4d). These results showed that rPoMSP-8 could induce immune response in mice and the mice anti-rPoMSP8 antibodies could recognize the rPoMSP-8s.

### Immune responses against rPoMSP-8 in mice

**Fig. 5:**
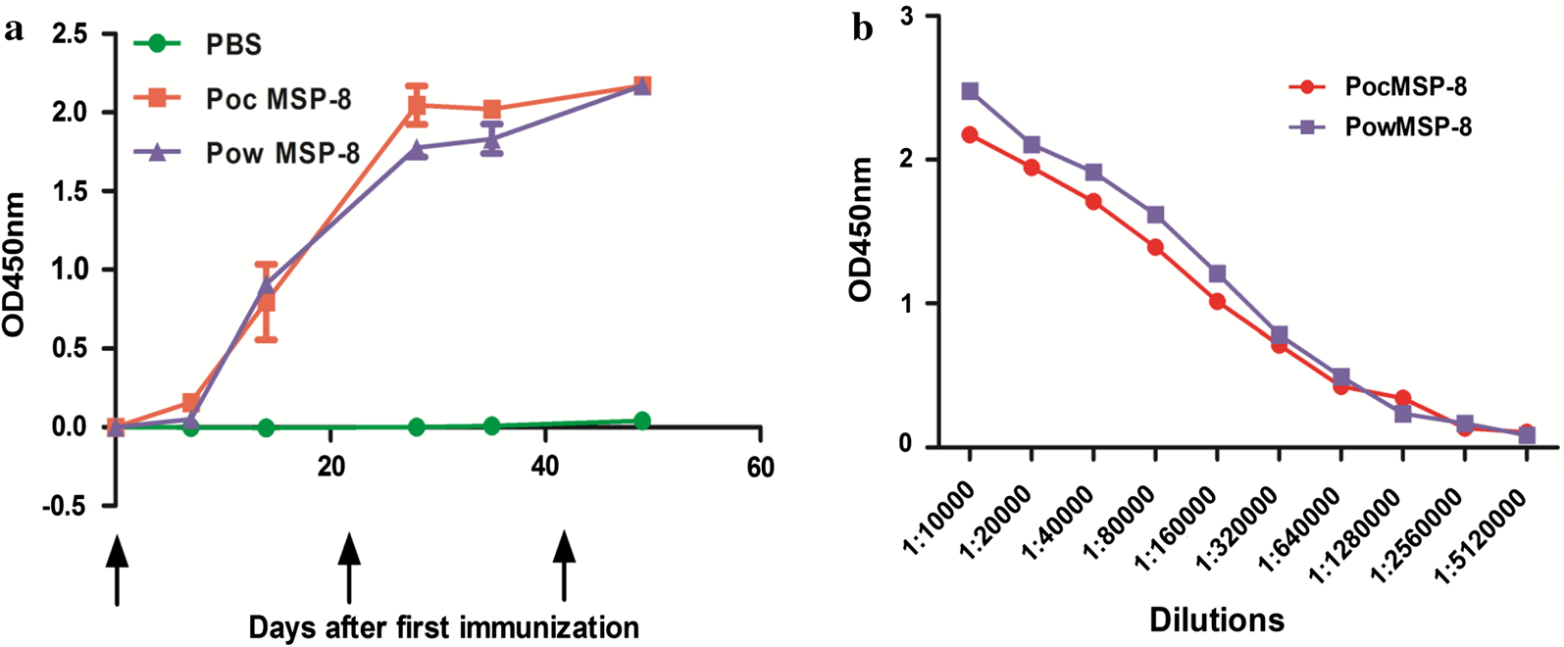
Immune responses in PoMSP-8-immunized mice. **a** IgG levels in PoMSP-8-immunized mice. IgG was detected at day 7 post-immunization, and the levels increased throughout the whole immunization period. **b** Data are presented as the geometric mean OD obtained at different concentrations, expressed as the reciprocal of the serum dilution obtained from mice immunized with PocMSP-8 or PowMSP-8 (dilutions from 1:10,000 to 1:5,120,000). Numbers on the X-axis indicate the dilutions tested. Antigen specificity was confirmed using pre-immune serum samples as a control

To begin characterizing immune responses generated by immunization with PoMSP-8, groups of 4 BALB/c mice were immunized with rPocMSP-8, rPowMSP-8, or PBS as a control, and the resultant antibody responses were measured by ELISA using the same recombinant proteins as the solid-phase coating antigen. The results provided in Fig. 5 show that both recombinants were immunogenic. ELISA results showed that antibodies against PoMSP-8 were detected one week after the primary booster. A high response was detected at day 14 post-immunization and continued to rise until day 28 post-immunization (Fig. 5a).

The mean serum antibody titers were evaluated 49 days after first immunization by ELISA. Similarly, PocMSP-8 and PowMSP-8 were linearly detected in ELISAs, and the PocMSP-8 and PowMSP-8 antigens could induce comparable IgG end-point titers (1:5,120,000; Fig. 5b).

In this study, high-avidity IgG antibodies were induced in all mouse groups immunized with adjuvant formulations. A higher IgG avidity response was detected in mice immunized with PocMSP-8 (mean: 94.84%) than was detected in mice immunized with PowMSP-8 (mean: 94.89%), but this difference was not statistically significant (*P* > 0.05).

## Discussion

Investigations into the extent of sequence variation amongst malaria vaccine candidates are undoubtedly important as the basis of effective malaria vaccine development. Here, we analyzed 42 clinical isolates from imported cases from Africa. The full length of *pomsp8* sequences (24 *pocmsp8* isolates and 18 *powmsp8* isolates) were completely conserved. The high degree of conservation observed among samples from 15 different African countries suggests that *pomsp8* may be useful in PCR-based diagnostic testing for *P. ovale*. This result is consistent with other reports in terms of MSP-8 sequence conservation in *P. falciparum*, *P. vivax* and *P. yoelii* [19, 20]. These findings suggest that the degree of conservation could be the result of maintained functions during red blood cell invasion and/or that these domains perform related or redundant roles in the parasite’s life-cycle [23, 24].

The *msp8* sequences placed *P. ovale curtisi* and *P. ovale wallikeri* in a distinct bifurcating branch in the phylogenetic tree and the results support an ancient time of divergence for the malarial parasite lineage [25]. This phylogenetic arrangement clearly indicates that *P. malariae* forms an outgroup both to rodent-infective species and to a primate-infective clade that includes *P. vivax* [26]. Whereas we found no conclusive evidence of selection acting on the polymorphism of *P. ovale*, likely due to the limited sample size, our comparative analyses based on phylogenetic methods indicate that *msp8* genes diverged under strong negative (purifying) selection, indicating functional constrains. The evidence that these proteins were conserved due to the potential action of purifying selection during their evolutionary history provides additional support for their consideration in malaria vaccine formulations.

Polymorphisms and immunogenicity are both within the scope of consideration when screening for new preclinical vaccine candidates. It had been reported that conserved regions were less antigenic and immunogenic than polymorphic regions [27–29]. Interestingly, the high conservation seen in PoMSP-8 mice was associated with a high level of immunogenicity, as both antibody titers were 1:5,120,000. This fragment of MSP-8 was expressed on the merozoite surface through a GPI-anchor and is taken up into erythrocytes [30]. GPI-anchors were previously found to be potent agonists of the toll-like receptor, as were adjuvants that facilitate immune responses [31]. As a surface protein, MSP-8 was exposed to the host immune system.

Antibodies play essential roles in protecting against blood-stage malaria. In this study, sera from mice immunized showed positive reactivity with rPocMSP-8 and rPowMSP-8, which are recombinant proteins. The fact that PoMSP-8 elicited humoral immune responses supports the conclusion that the conserved sequences contain B epitopes. Cross-reaction between PocMSP-8 and PowMSP-8 was detected by both ELISA and western immunoblot analyses. These findings suggested that rPoMSP-8 shares similar and conserved antigenic determinants and that a complex network of cross-reactivity exists between *P. ovale* sp. The finding may explain the diversity between different species and enable measurements of species-specific efficacy in vaccine trials.

Production of high-avidity antibodies is essential for preventing severe disease during malaria infections. Our results indicated that both PocMSP-8 and PowMSP-8 induced high avidity antibodies (PocMSP-8: 94.84%, PowMSP-8: 92.69%). High-quality antibodies represent an important factor involved in preventing and protecting against infections [32]. These findings suggest that MSP-8 in mice is a valid model for understanding the immune response and may compensate for the difficulty of culturing this parasite *in vitro*. As such, it warrants further study.

## Conclusions

The findings of this study demonstrate the remarkable conservation and high immunogenicity of both PoMSP-8s. Cross-reaction between PocMSP-8 and PowMSP-8 was detected, suggesting that immune responses may be mediated by a repertoire of antibodies to common epitopes between both *P. ovale* subspecies. These data provide valuable reference information regarding the genetic diversity and immunogenicity of *P. ovale curtisi* and *P. ovale wallikeri* isolates imported from Africa to China. Therefore, PoMSP-8 may potentially serve as a candidate for malaria vaccine design, although further evaluation needs be carried out to validate its potential and limitations.

## Additional files

**Additional file 1: Table S1.** Information on imported *P. ovale curtisi* and *P. ovale wallikeri*.

**Additional file 2: Figure S1.** Amino acid sequence alignment of *P. ovale* MSP-8 full length. **a** *P. ovale curtisi*. **b** *P. ovale wallikeri*.

**Additional file 3: Table S2.** The *msp8* Gene ID number of other *Plasmodium* species.

## Abbreviations

MSP-8: merozoite surface protein-8
rPoMSP-8: recombinant PoMSP-8
GPI: glycosylphosphatidylinositol
PCR: polymerase chain reaction
aa: amino acid
EGF: epidermal growth factor
ELISA: enzyme-linked immunosorbent assays
AI: avidity index
