# Supplementary material for: Immunogenicity analysis of genetically conserved segments in Plasmodium ovale merozoite surface protein-8
Source: Parasit Vectors. 2019 Apr 11;12:164. doi: 10.1186/s13071-019-3412-0 (PMC6460738; doi:10.1186/s13071-019-3412-0)
Supplement: Supplementary file 1 — Additional file 1: Table S1. Information on imported P. ovale curtisi and P. ovale wallikeri. [file 13071_2019_3412_MOESM1_ESM.pdf]

Table S1 Information of imported *P.ovale curtisi* and *P.ovale wallikeri*

Species confirmation	Isolate number	Origin of country	Parasitaemia
P.ovale curtisi	Poc-1	Angola	9377
	Poc-3	Angola	15351
	Poc-6	Angola	4490
	Poc-8	Angola	8727
	Poc-14	Angola	5922
	Poc-21	Equatorial Guinea	3477
	Poc-27	Equatorial Guinea	8834
	Poc-33	Equatorial Guinea	32000
	Poc-34	Equatorial Guinea	11025
	Poc-40	Equatorial Guinea	9276
	Poc-46	Republic of Congo	16753
	Poc-52	Republic of Congo	3830
	Poc-55	Republic of Congo	8076
	Poc-58	Guinea	2200
	Poc-59	Ghana	4053
	Poc-60	Gabon	9363
	Poc-62	Cameroon	2736
	Poc-64	Cameroon	3800
	Poc-71	Niger	2500
	Poc-72	Nigeri	5350
	Poc-76	Nigeri	4163
	Poc-78	Nigeri	6000
	Poc-81	Nigeri	3651
	Poc-86	Zambia	5424
P.ovale wallikeri	Pow-1	Angola	1893
	Pow-3	Angola	10971
	Pow-7	Angola	34793
	Pow-14	Angola	1837
	Pow-18	Angola	22118
	Pow-26	Equatorial Guinea	645
	Pow-37	Equatorial Guinea	1934
	Pow-39	Equatorial Guinea	4500
	Pow-40	Equatorial Guinea	6200
	Pow-49	Equatorial Guinea	11538
	Pow-55	Republic of Congo	4500
	Pow-63	Guinea	3289
	Pow-66	Gabo	6818
	Pow-70	Cameroon	7384
	Pow-73	Cote d'Ivoire	4850
	Pow-74	Liberia	8380
	Pow-76	Mozambique	2111
	Pow-80	Nigeria	3613
