# Supplementary material for: Immunogenicity analysis of genetically conserved segments in Plasmodium ovale merozoite surface protein-8
Source: Parasit Vectors. 2019 Apr 11;12:164. doi: 10.1186/s13071-019-3412-0 (PMC6460738; doi:10.1186/s13071-019-3412-0)
Supplement: Supplementary file 2 — Additional file 2: Figure S1. Amino acid sequence alignment of P. ovale MSP-8 full length. a P. ovale curtisi. b P. ovale wallikeri. [file 13071_2019_3412_MOESM2_ESM.pdf]

a

```
PocMSP8 MVMIMKKNSLILIFLIFSLIYYKNTVEGNIDIANGNGIININNNDSNGGNGSDKKIPPMNTGGNNDNMNKNNNVGGNDNGNNVDDLTNNSLKDDSKDNDENKNKKENND
Poc(1)  .............................................................................................................
Poc(3)  .............................................................................................................
Poc(6)  .............................................................................................................
Poc(8)  .............................................................................................................
Poc(14) .............................................................................................................
Poc(21) .............................................................................................................
Poc(27) .............................................................................................................
Poc(33) .............................................................................................................
Poc(34) .............................................................................................................
Poc(40) .............................................................................................................
Poc(46) .............................................................................................................
Poc(52) .............................................................................................................
Poc(55) .............................................................................................................
Poc(58) .............................................................................................................
Poc(59) .............................................................................................................
Poc(60) .............................................................................................................
Poc(62) .............................................................................................................
Poc(64) .............................................................................................................
Poc(71) .............................................................................................................
Poc(72) .............................................................................................................
Poc(76) .............................................................................................................
Poc(78) .............................................................................................................
Poc(81) .............................................................................................................
Poc(86) .............................................................................................................

PocMSP8 IESKGDDNDNKEDNNSEKNDESLKKILNIVDEMENIQELLDGDSNILDKYNIKLVDEEDGDSKKKKLIGEYDLKMIKKVLLFREKISRTCENNFMNVNLTLKKCFNKDD
Poc(1)  .............................................................................................................
Poc(3)  .............................................................................................................
Poc(6)  .............................................................................................................
Poc(8)  .............................................................................................................
Poc(14) .............................................................................................................
Poc(21) .............................................................................................................
Poc(27) .............................................................................................................
Poc(33) .............................................................................................................
Poc(34) .............................................................................................................
Poc(40) .............................................................................................................
Poc(46) .............................................................................................................
Poc(52) .............................................................................................................
Poc(55) .............................................................................................................
Poc(58) .............................................................................................................
Poc(59) .............................................................................................................
```

```
Poc(60) .............................................................................................................
Poc(62) .............................................................................................................
Poc(64) .............................................................................................................
Poc(71) .............................................................................................................
Poc(72) .............................................................................................................
Poc(76) .............................................................................................................
Poc(78) .............................................................................................................
Poc(81) .............................................................................................................
Poc(86) .............................................................................................................

PocMSP8 PKLSKSCEKIKRGLSKNNMSIEDFILGLLEDLFDKINDNFIQNDSFDLNDYLADFELINYLLLHESAELLKEIIHILDAINFKVESDALTKISNSAYSGMNINDKIKDD
Poc(1)  .............................................................................................................
Poc(3)  .............................................................................................................
Poc(6)  .............................................................................................................
Poc(8)  .............................................................................................................
Poc(14) .............................................................................................................
Poc(21) .............................................................................................................
Poc(27) .............................................................................................................
Poc(33) .............................................................................................................
Poc(34) .............................................................................................................
Poc(40) .............................................................................................................
Poc(46) .............................................................................................................
Poc(52) .............................................................................................................
Poc(55) .............................................................................................................
Poc(58) .............................................................................................................
Poc(59) .............................................................................................................
Poc(60) .............................................................................................................
Poc(62) .............................................................................................................
Poc(64) .............................................................................................................
Poc(71) .............................................................................................................
Poc(72) .............................................................................................................
Poc(76) .............................................................................................................
Poc(78) .............................................................................................................
Poc(81) .............................................................................................................
Poc(86) .............................................................................................................

PocMSP8 ITNLLKMPSAKFFKIGIDKKTKMLIPVQAQHKGSSMKQFAYHFLDKNKVCEHTKCPLNSNCYVINSEETCRCLPGFSDVKIDNVMNCVRDDTMDCSNNNGGCDVNATCS
Poc(1)  .............................................................................................................
Poc(3)  .............................................................................................................
Poc(6)  .............................................................................................................
Poc(8)  .............................................................................................................
Poc(14) .............................................................................................................
Poc(21) .............................................................................................................
```

```
Poc(27) .............................................................................................................
Poc(33) .............................................................................................................
Poc(34) .............................................................................................................
Poc(40) .............................................................................................................
Poc(46) .............................................................................................................
Poc(52) .............................................................................................................
Poc(55) .............................................................................................................
Poc(58) .............................................................................................................
Poc(59) .............................................................................................................
Poc(60) .............................................................................................................
Poc(62) .............................................................................................................
Poc(64) .............................................................................................................
Poc(71) .............................................................................................................
Poc(72) .............................................................................................................
Poc(76) .............................................................................................................
Poc(78) .............................................................................................................
Poc(81) .............................................................................................................
Poc(86) .............................................................................................................

PocMSP8 LIDKKIVCECKENFEGDGIYCSNSILNSINCFIFLIIVMLCLYLL  481
Poc(1)  .............................................  481
Poc(3)  .............................................  481
Poc(6)  .............................................  481
Poc(8)  .............................................  481
Poc(14) .............................................  481
Poc(21) .............................................  481
Poc(27) .............................................  481
Poc(33) .............................................  481
Poc(34) .............................................  481
Poc(40) .............................................  481
Poc(46) .............................................  481
Poc(52) .............................................  481
Poc(55) .............................................  481
Poc(58) .............................................  481
Poc(59) .............................................  481
Poc(60) .............................................  481
Poc(62) .............................................  481
Poc(64) .............................................  481
Poc(71) .............................................  481
Poc(72) .............................................  481
Poc(76) .............................................  481
Poc(78) .............................................  481
Poc(81) .............................................  481
```

```
Poc(86)  .............................................     481
```

b

```
PowMSP8  MVMIMKKNSLILIFLIFSLIYYKNTVEGNIDIANGNGIININNNDSNGGNGSDKKIPPMNTGGNNDNMNKNNNVGGNDNGNNVDDLTNNSLKDDSKDNDENKNKKENND
Pow(1)   .............................................................................................................
Pow(3)   .............................................................................................................
Pow(7)   .............................................................................................................
Pow(14)  .............................................................................................................
Pow(18)  .............................................................................................................
Pow(26)  .............................................................................................................
Pow(37)  .............................................................................................................
Pow(39)  .............................................................................................................
Pow(40)  .............................................................................................................
Pow(49)  .............................................................................................................
Pow(55)  .............................................................................................................
Pow(63)  .............................................................................................................
Pow(66)  .............................................................................................................
Pow(70)  .............................................................................................................
Pow(73)  .............................................................................................................
Pow(74)  .............................................................................................................
Pow(76)  .............................................................................................................
Pow(80)  .............................................................................................................

PowMSP8  IESKGDDNDNKEDNNSEKNDESLKKILNIVDEMENIQELLDGDSNILDKYNIKLVDEEDGDSKKKKLIGEYDLKMIKKVLLFREKISRTCENNFMNVNLTLKKCFNKDD
Pow(1)   .............................................................................................................
Pow(3)   .............................................................................................................
Pow(7)   .............................................................................................................
Pow(14)  .............................................................................................................
Pow(18)  .............................................................................................................
Pow(26)  .............................................................................................................
Pow(37)  .............................................................................................................
Pow(39)  .............................................................................................................
Pow(40)  .............................................................................................................
Pow(49)  .............................................................................................................
Pow(55)  .............................................................................................................
Pow(63)  .............................................................................................................
Pow(66)  .............................................................................................................
Pow(70)  .............................................................................................................
Pow(73)  .............................................................................................................
Pow(74)  .............................................................................................................
Pow(76)  .............................................................................................................
Pow(80)  .............................................................................................................
```

```
PowMSP8  PKLSKSCEKIKRGLSKNNMSIEDFILGLLEDLFDKINDNFIQNDSFDLNDYLADFELINYLLLHESAELLKEIIHILDAINFKVESDALTKISNSAYSGMNINDKIKDD
Pow(1)   .............................................................................................................
Pow(3)   .............................................................................................................
Pow(7)   .............................................................................................................
Pow(14)  .............................................................................................................
Pow(18)  .............................................................................................................
Pow(26)  .............................................................................................................
Pow(37)  .............................................................................................................
Pow(39)  .............................................................................................................
Pow(40)  .............................................................................................................
Pow(49)  .............................................................................................................
Pow(55)  .............................................................................................................
Pow(63)  .............................................................................................................
Pow(66)  .............................................................................................................
Pow(70)  .............................................................................................................
Pow(73)  .............................................................................................................
Pow(74)  .............................................................................................................
Pow(76)  .............................................................................................................
Pow(80)  .............................................................................................................

PowMSP8  ITNLLKMPSAKFFKIGIDKKTKMLIPVQAQHKGSSMKQFAYHFLDKNKVCEHTKCPLNSNCYVINSEETCRCLPGFSDVKIDNVMNCVRDDTMDCSNNNGGCDVNATCS
Pow(1)   .............................................................................................................
Pow(3)   .............................................................................................................
Pow(7)   .............................................................................................................
Pow(14)  .............................................................................................................
Pow(18)  .............................................................................................................
Pow(26)  .............................................................................................................
Pow(37)  .............................................................................................................
Pow(39)  .............................................................................................................
Pow(40)  .............................................................................................................
Pow(49)  .............................................................................................................
Pow(55)  .............................................................................................................
Pow(63)  .............................................................................................................
Pow(66)  .............................................................................................................
Pow(70)  .............................................................................................................
Pow(73)  .............................................................................................................
Pow(74)  .............................................................................................................
Pow(76)  .............................................................................................................
Pow(80)  .............................................................................................................

PowMSP8  LIDKKIVCECKENFEGDGIYCSNSILNSINCFIFLIIVMLCLYLL  481
Pow(1)   .............................................  481
Pow(3)   .............................................  481
```

Pow(7) 481
Pow(14) 481
Pow(18) 481
Pow(26) 481
Pow(37) 481
Pow(39) 481
Pow(49) 481
Pow(55) 481
Pow(63) 481
Pow(66) 481
Pow(70) 481
Pow(73) 481
Pow(74) 481
Pow(76) 481
Pow(80) 481
