# Supplementary material for: Immunogenicity analysis of genetically conserved segments in Plasmodium ovale merozoite surface protein-8
Source: Parasit Vectors. 2019 Apr 11;12:164. doi: 10.1186/s13071-019-3412-0 (PMC6460738; doi:10.1186/s13071-019-3412-0)
Supplement: Supplementary file 3 — Additional file 3: Table S2. The msp8 Gene ID number of other Plasmodium species. [file 13071_2019_3412_MOESM3_ESM.pdf]

Table S2 The *msp8* Gene ID number of other *Plasmodium* species

Species	Strain	Gene ID from PlasmoDB
P. falciparum	3D7	PF3D7_0502400
	IT	PFIT_0502500
P. malariae	UG01	PmUG01_06023900
P. vivax	P01	PVP01_1032600
	Sal-1	PVX_097625
P. reichenowi	CDC	PRCDC_0501700
	G01	PRG01_0501600
P. knowlesi	strain H	PKNH_1031500
	strain Malayan Strain Pk1 A	PKNOH_S07469500
P. cynomolgi	strain B	PCYB_104050
	strain M	PcyM_1033300
P. fragile	strain nilgiri	AK88_01475
P. coatneyi	Hackeri	PCOAH_00031550
P. relictum	SGS1-like	PRELSG_1030100
P. yoelii	17X	PY17X_1103300
	17XNL	PY06415
P. chabaudi	chabaudi	PCHAS_1101900
P. berghei	ANKA	PBANKA_1102200
P. vinckei	petteri strain CR	YYG_01170
	vinckei strain vinckei	YYE_02689
